# Acute Hemodynamic Changes Induced by Cardiac Contractility Modulation Evaluated Using the NICaS^®^ System: A Pilot Study

**DOI:** 10.3390/jcm14072172

**Published:** 2025-03-22

**Authors:** Andrea Madeo, Silvana De Bonis, Anna Lucia Cavaliere, Giovanni Bisignani

**Affiliations:** 1 Cardiology Department, Ferrari Hospital, ASP Cosenza, 87012 Castrovillari, Italy; giovanni.bisignani@aspcs.it; 2 Cardiology Department, Giannetasio Hospital, ASP Cosenza, 87064 Rossano, Italy; silvanadebonis68@gmail.com

**Keywords:** cardiac contractility modulation, NICaS^®^, heart failure, hemodynamics, non-invasive monitoring

## Abstract

**Background/Objectives**: Heart failure (HF) with reduced ejection fraction remains a significant global health challenge despite advances in medical therapy. Cardiac contractility modulation (CCM) is a promising treatment for symptomatic HF patients who are ineligible for cardiac resynchronization therapy (CRT). Non-invasive methods to assess the acute hemodynamic effects of CCM are critical to optimize care and guide treatment. This study aimed to evaluate the acute impact of CCM on stroke volume (SV) and total peripheral resistance index (TPRI) using the non-invasive bioimpedance-based system (NICaS^®^). **Methods**: Eight HF patients (median age: 64.6 years, median left ejection fraction (LVEF): 34.5%) underwent implantation of the Optimizer Smart Mini CCM device. Hemodynamic parameters, including SV and TPRI, were measured using NICaS^®^ at baseline (pre-implantation) and at 1 week, 1 month, and 3 months post-implantation. Measurements were repeated eight times per session and analyzed using non-parametric statistical tests, including the Kruskal–Wallis test, Mann–Whitney test, and Kolmogorov–Smirnov test. **Results**: Median SV increased significantly from 40.02 mL (interquartile range (IQR): 32.62–78.16 mL) at baseline to 69.83 mL (IQR: 58.63–86.36 mL) at 3 months (*p* < 0.0001). Median TPRI decreased significantly from 2537 dn s/cm^5^ m^2^ (IQR: 1807–3084 dn s/cm^5^ m^2^) to 1307 dn s/cm^5^ m^2^ (IQR: 1119–1665 dn s/cm^5^ m^2^) over the same period (*p* < 0.0001). CCM therapy significantly improved SV and reduced TPRI in HF patients within three months of implantation. **Conclusions**: NICaS^®^ provided a reliable, non-invasive tool for monitoring these acute hemodynamic changes, supporting its use in clinical practice.

## 1. Introduction

Heart failure (HF) remains a leading cause of morbidity and mortality worldwide, despite significant advances in pharmacological management. Many patients experience persistent symptoms such as fatigue, dyspnea, and peripheral edema, resulting in reduced quality of life and frequent hospitalizations [1]. In patients with chronic HF characterized by reduced left ventricular ejection fraction (LVEF) and persistent symptoms despite optimal medical therapy (OMT), cardiac contractility modulation (CCM) therapy offers a promising alternative. This advanced therapy is particularly suited for patients with LVEF between 25 and 50% who are ineligible for cardiac resynchronization therapy (CRT) due to narrow QRS complexes [2].

CCM delivers high-energy (7.5 V) non-excitatory electrical impulses during the absolute refractory period, enhancing myocardial contractility without increasing oxygen demand [3]. Clinical studies have demonstrated improvements in exercise capacity, quality of life, and functional status in HF patients treated with CCM [4]. These benefits are assessed through a variety of clinical, functional, and imaging parameters, including LVEF, peak oxygen consumption (VO_2_ max), and New York Heart Association (NYHA) classification. Additional measures, such as six-minute walk tests, patient-reported outcomes (e.g., the Kansas City Cardiomyopathy Questionnaire), and biomarkers like NT-proBNP, further aid in evaluating therapeutic efficacy. Although the established long-term benefits of CCM have been evaluated, there is a critical need for non-invasive methods to assess its acute hemodynamic effects. While invasive techniques, such as cardiac catheterization, provide accurate measurements, their associated risks and procedural complexity limit widespread use [5]. Conversely, non-invasive tools enable safer, repeatable, and dynamic monitoring of CCM impact on cardiac performance. This study aims to evaluate the acute effects of CCM therapy on stroke volume (SV) and total peripheral resistance index (TPRI) using the bioimpedance non-invasive monitoring system NICaS^®^. This portable, non-invasive device leverages bioimpedance technology to monitor cardiac output and fluid status, potentially guiding clinical decision-making and improving outcomes for HF patients.

## 2. Materials and Methods

### 2.1. Patient Poulation

All patients enrolled in the study between September 2023 and September 2024 provided written informed consent. The study was conducted according to the Declaration of Helsinki and was approved by the ethics committee of ASP Cosenza with protocol number 24110/2023. Pharmacological therapy was optimized for all participants following the European Society of Cardiology (ESC) guidelines for heart failure (HF) (Table 1). Inclusion criteria required a history of at least two hospitalizations due to HF exacerbations. Furthermore, all patients were offered CCM therapy to improve their quality of life and consented to undergo device implantation. The median age of patients was 64.5 years (IQR: 50–78 years). Five patients had dilated cardiomyopathy, while three had ischemic cardiomyopathy. The median LVEF was 34.5% (IQR: 28–45%) (Table 1). All patients exhibited severe left ventricular (LV) dysfunction and carried an implantable cardioverter defibrillator (ICD).

### 2.2. CCM Implantation

The CCM device used in this study was the (OPTIMIZER Smart Mini Implantable Pulse Generator (IPG), Impulse Dynamics (Marlton, NJ, USA). The device was implanted subcutaneously in the pectoral region of the right chest. Two right ventricular leads were positioned 2 cm apart within the intra-ventricular septum to deliver CCM therapy. Fluoroscopic guidance was used during lead placement to ensure proper positioning and avoid complications, such as lead migration or perforation. Special attention was taken to maintain sufficient separation between the CCM leads and ICD leads to minimize interference. Once lead placement was confirmed, the leads were connected to the IPG, which was tested for pacing thresholds, sensing capabilities, and proper therapy delivery. The IPG was inserted into a subcutaneous pocket created in the pectoral region. Following the placement, the pocket was closed, and device functionality was verified. Patients were monitored postoperatively to ensure proper lead positioning, cardiac function, and overall sufficient device therapy delivery.

### 2.3. NICaS^®^ Measurements

The NICaS^®^ was utilized to non-invasively measure and analyze cardiovascular parameters through bioimpedance technology. This system utilizes a tetrapolar configuration, transmitting a 1.4 mA alternating current at a frequency of 32 kHz through electrodes positioned on both wrists of the patients. The electrodes were linked to the hardware device, which was subsequently connected to a laptop for data acquisition. The NICaS^®^ software (v. 3.63.15) interface facilitated patient data management and guided the operator through the process for recording electrical impedance changes caused by the systolic expansion of arteries. These impedance variations were captured, amplified, filtered within the device, and transmitted to a microprocessor where they were digitized and analyzed through proprietary algorithms. The system calculated key cardiovascular metrics such as SV and TPRI. Upon completing each session, the software generated comprehensive reports for analysis, and the data could be exported in various formats for further evaluation. Measurements were taken one day before the implantation of the CCM device, one week, one month, and three months following the implant. Each measurement was repeated 8 times. The analysis of the measurements focused on the values of SV (mL) and TPRI (dn s/cm^5^m^2^), which provide insights into the pumping ability of the heart and the load against which the heart must work, respectively. Both SV and TPRI measurements were taken eight times at four time points (baseline, 1 week post-implantation, 1 month post-implantation, and 3 months post-implantation) for each of the eight patients, with a total of 256 datapoints. We also assessed SV using standard Doppler echocardiography by comparing the median of the eight NICaS measurements taken at each visit (baseline, one week, one month, and three months) with the SV value measured by the Vivid E80 Echocardiography system (GE Healthcare^®^, Chicago, IL, USA) on the same visit. Data were exported as csv files and then imported on the Prism Analysis Software (v. 10.4.1) to process graphs, create descriptive statistics, and perform the Kruskal–Wallis test, followed by a post hoc analysis to compare the distributions of SV and TPRI at the four different measurement times. The Mann–Whitney *U* test was used to determine which specific time points differ in median while the Kolmogorov–Smirnov test was used to compare the entire distribution between two independent groups of values at different measurement times.

## 3. Results

**Figure 1:**
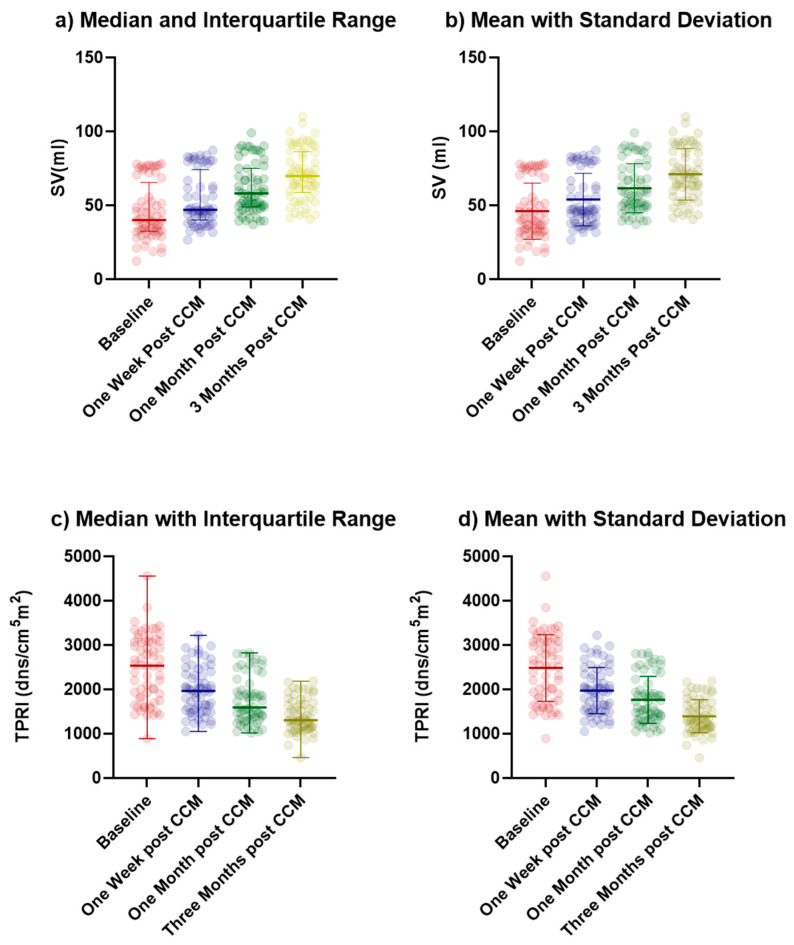
Descriptive statistics of changes in SV (**a**,**b**) and TPRI (**c**,**d**) across four time points, presented as (**a**,**c**) median with interquartile range and (**b**,**d**) mean with standard deviation. The points correspond to each of the eight measurements taken for each of the eight patients.

The SV increased significantly over the four measurement time points (Figure 1a). At baseline, the median SV was 40.02 mL (IQR: 32.62–78.16 mL), which rose to 46.92 mL (IQR: 40.9–74.23 mL) one week after CCM implantation, representing a substantial improvement. This trend continued after one month from implantation, with a median SV of 58.04 mL (IQR: 48.98–75.04 mL), and further increased to 69.83 mL (IQR: 58.63–86.36 mL) at the three-month follow-up. On the other hand, the TPRI distribution followed a downward trend. As a matter of fact, at baseline the median for TPRI was 2537 dn s/cm^5^ m^2^ (IQR: 1807–3084 dn s/cm^5^ m^2^). By 1-week post-implantation, this value had significantly decreased to 1966 dn s/cm^5^ m^2^ (IQR of 1532–2340 dn s/cm^5^ m^2^). Further reductions were observed at 1 month and 3 months, with the median TPRI decreasing to 1594 dn s/cm^5^ m^2^ (IQR 1334–2096 dn s/cm^5^ m^2^) and 1307 dn s/cm^5^ m^2^ (IQR 1119–1665 dn s/cm^5^ m^2^), respectively (Figure 1c).

**Figure 2:**
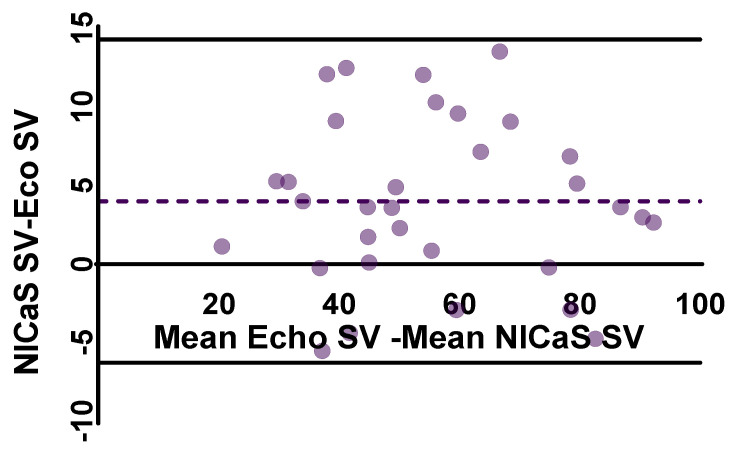
Bland–Altman plot illustrating the agreement between NICaS^®^ and echocardiography stroke volume measurements. Each point corresponds to a single paired measurement of stroke volume from NICaS^®^ and echocardiography. *X*-axis value is the average of the two measurements for a given observation. *Y*-axis value is the difference between NICaS^®^ and echocardiography measurements. The dashed purple line shows the mean difference or bias (4.018 mL). Points above the line indicate that NICaS measured a higher stroke volume than echocardiography for those observations. The black lines indicate the limits of agreement.

**Figure 3:**
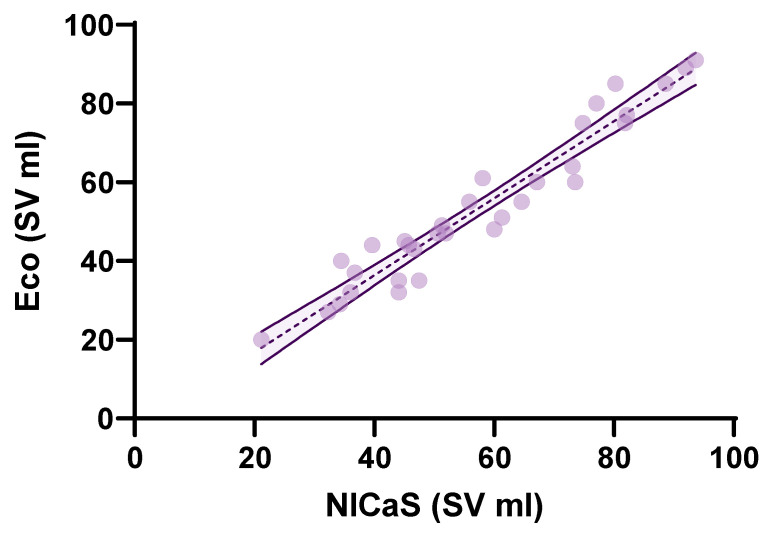
Scatter plot with linear regression analysis comparing NICaS^®^ and Echocardiography SV measurements.

Statistical analysis using the Kruskal–Wallis test revealed a significant difference in SV distributions across all the measurements (*H* = 57.41 *p* < 0.0001). Pairwise comparisons using the Mann–Whitney *U* test revealed statistically significant increases in SV between baseline, one week, one month, and three months, highlighting a consistent upward trend. The Kolmogorov–Smirnov test further confirmed significant differences in the distribution of SV values across all time points, indicating not only changes in median values but also shifts in the overall distribution (Table 2). As in the case of SV, the Kruskal–Wallis test showed a significant difference in TPRI distributions across all the measurements (*H* = 82.42 *p* < 0.0001). Median comparisons using the Mann–Whitney *U* test revealed a statistically significant decrease in TPRI between baseline, one week, one month, and three months. Finally, the Kolmogorov–Smirnov test further confirmed significant differences in the distribution of TPRI values across all time points (Table 2). The comparison between NICaS SV and echocardiography SV revealed a controlled difference of 3–4 mL between the two methods. The mean stroke volume for bioimpedance was 57.67 mL (95% CI: 50.93 to 64.42) across all the time points, while the mean stroke volume for echocardiography was 53.66 mL (95% CI: 46.82 to 60.50). Bland–Altman analysis further confirmed a systematic bias, with bioimpedance overestimating stroke volume by 4.02 mL (Figure 2). The limits of agreement (LOA) indicates that while bioimpedance and echocardiography are strongly correlated, some variability exists, which may stem from biological differences, measuring noise, or inherent methodological discrepancies between the two techniques. A linear regression model was also applied. The scatter plot with regression analysis (Figure 3) demonstrates a strong correlation (R^2^ = 0.930). The *p*-value for the regression model was <0.0001, confirming statistical significance (Figure 3).

## 4. Discussion

Non-invasive systems like NICaS^®^ offer a safer and more practical alternative to invasive hemodynamic monitoring methods such as Swan-Ganz catheterization which is considered the gold standard and presents several complications including bleeding, infection, and vascular injury. In one significant study by Tanino et al., NICaS^®^ was used to monitor chronic heart failure patients, and its derived parameters (SV and TPRI) were compared with conventional measures, including left ventricular ejection fraction (LVEF) and B-type natriuretic peptide (BNP) levels [6]. The NICaS^®^-derived parameters were highly correlated with traditional measurements (*p* < 0.001). Importantly, NICaS^®^ also predicted heart failure readmission with clinically relevant threshold values which closely aligned with values from invasive monitoring techniques like Swan-Ganz. These findings underscore NICaS^®^’s ability to provide meaningful, actionable data in outpatient follow-up, without the risks of invasive catheterization. Similarly, in a study by Lavie et al., NICaS^®^ and the Swan-Ganz catheter were compered to measure cardiac output (CO) and systemic vascular resistance (SVR) in pregnant women with preeclampsia [7]. NICaS^®^ consistently identified lower CO and higher SVR in pre-eclamptic patients compared to normotensive controls. These measurements mirrored the findings from Swan-Ganz catheterization, with CO peaking during delivery and declining postpartum. The strong correlation between the two methods in this study further validated NICaS^®^ as a non-invasive alternative in high-risk obstetric cases, where invasive monitoring poses additional complications. A broader comparison of NICaS^®^ and Swan-Ganz demonstrated a mean bias of ±0.22 L/min when compared to Swan-Ganz for measuring CO, with limits of agreement ranging between −1.05 L/min and +0.79 L/min [8]. These limits are well within the acceptable clinical range, particularly in the context of acute care where rapid and reliable measurements are critical. The study found a significant correlation (*r* = 0.87) between NICaS^®^ and Swan-Ganz, highlighting the system’s precision in capturing real-time changes in CO. This high level of agreement confirmed that NICaS^®^ can replicate the accuracy of thermodilution methods while offering the advantages of non-invasive monitoring. In a detailed evaluation by Goedje et al., NICaS^®^ was calibrated against arterial thermodilution methods to assess its performance in cardiac surgery patients [9]. NICaS^®^ showed a bias of 0.07 L/min compared to Swan-Ganz and maintained a high level of precision over 24 h of continuous monitoring. The study demonstrated that NICaS^®^ effectively tracks CO even during substantial fluctuations in vascular tone and hemodynamic status, such as those induced by vasoactive drugs. Limits of agreement ranged from −1.68 L/min to +2.32 L/min, further validating the reliability of NICaS^®^ in dynamic clinical environments. Importantly, after initial calibration, NICaS^®^ provided consistent monitoring without the need for repeated invasive calibrations, underscoring its efficiency and ease of use. These studies collectively confirmed the scientific validity and clinical reliability of NICaS^®^. NICaS^®^ provided precise, non-invasive hemodynamic data with strong correlations to the gold-standard Swan-Ganz catheter and biases consistently near zero. Therefore, the system can be considered an invaluable tool in both acute and chronic care settings, particularly when invasive monitoring poses risks or is impractical. NICaS^®^ offers the distinct advantage of continuous, non-invasive monitoring, enabling real-time decision-making without the complications associated with catheter-based methods. Moreover, its reproducibility in the hemodynamic monitoring of HF patients is very useful for clinicians. This study provides evidence on the acute hemodynamic effects of CCM in HF patients with reduced LVEF, evaluated using the non-invasive NICaS^®^ system. The observed significant improvements in both SV and TPRI across multiple time points demonstrate that CCM delivers rapid, sustained cardiovascular benefits. These findings align with the concept of myocardial remodeling over time [10]. As demonstrated by Kuschyk et al. (2021), CCM not only improves LVEF, exercise capacity, and NYHA class, but also sustains these benefits over longer periods (up to 24 months) with reduced hospitalizations [11]. The acute improvements observed in this study reinforce that CCM beneficial effects on myocardial function begin immediately. Therefore, early intervention can result in rapid clinical benefits, which may alleviate symptoms such as fatigue and shortness of breath, typically experienced by HF patients. Previous work by Mohri et al. on Langendorff-perfused ferret hearts also supports the acute mechanism of CCM, showing that biphasic electrical stimulation during the absolute refractory period enhanced contractility independent of beta-adrenergic stimulation [12]. In this study, significant changes were observed in SV within a short time frame. These results are consistent with Mohri’s earlier findings, suggesting that the increased contractility seen with CCM is primarily due to its direct effects on myocardial performance and calcium handling.

A comprehensive individual patient data meta-analysis by Giallauria et al., published in ESC Heart Failure, analyzed data from 801 patients across five trials and found that, compared with control groups, CCM therapy significantly improved peak oxygen consumption (peak VO_2_) by a mean difference of +0.93 mL/kg/min (95% CI: 0.56–1.30, *p* < 0.00001) and increased the 6-min walk test (6MWT) distance by +17.97 m (95% CI: 5.48–30.46, *p* = 0.005). Additionally, quality of life, assessed using the Minnesota Living with Heart Failure Questionnaire (MLWHFQ), improved by a mean reduction of −7.85 points (95% CI: −10.76 to −4.94, *p* < 0.00001), indicating that CCM therapy not only enhances exercise capacity but also meaningfully reduces symptoms [13]. The significant reduction in TPRI, from a median baseline of 2537 dns/cm^5^⋅m^2^ to 1394.89 dns/cm^5^⋅m^2^ by three months post-implantation can be explained as a consequence of improved energy efficiency and, consequently, enhanced systolic-diastolic myocardial performance leading to a secondary reduction in left ventricular afterload, which typically increases as a reactive response to chronic heart failure [10,11,12,13,14,15,16,17]. In addition, Masarone et al, demonstrated CCM-related improvements of global longitudinal strain (GLS) and mechano-energetic efficiency in patients with heart failure with reduced ejection fraction (HFrEF). These evidence highlighted the net clinical benefit of CCM therapy on global health status of the HF patients, including those with preserved ejection fraction [18,19].

Moreover, CCM may also play a role in reducing myocardial remodeling and arrhythmic burden. De Donno et al., described the case of a 65-year-old male with heart failure experiencing frequent ICD shocks due to sustained ventricular arrhythmias, despite being on maximal antiarrhythmic and heart failure therapy. Following CCM implantation, there was a complete cessation of sustained ventricular arrhythmias, with no ICD interventions recorded at the 6-month follow-up. Additionally, the patient’s left ventricular ejection fraction (LVEF) improved from 25% to 35%, with a 6MWT distance increasing from 360 m to 580 m as a sign of both symptomatic and structural benefits [14].

CCM improves calcium handling within cardiomyocytes by upregulating proteins such as sarco/endoplasmic reticulum calcium ATPase 2 (SERCA2) while reducing the inhibitory effects of phospholamban, leading to enhanced myocardial contractility and reverse remodeling. These findings suggest that, in addition to improving exercise tolerance, CCM may have an antiarrhythmic effect in selected patients, although further large-scale studies are needed to confirm this hypothesis. The European Society of Cardiology (ESC) guidelines recognize CCM as a potential treatment for patients with LVEF between 25% and 50%, QRS ≤ 130 ms and NYAH class III or IV despite OMT.

## 5. Conclusions

The findings from this study offer several important clinical implications. First, the rapid improvements in SV and reduction in TPRI suggest that CCM could be an effective early intervention in heart failure patients who are not receiving CRT and still experience symptoms despite OMT. The observed hemodynamic changes imply that CCM not only improves cardiac function but also reduces systemic vascular load, which is critical for the long-term management of heart failure. This highlights the potential of CCM in reducing hospitalization rates and improving quality of life for HF patients, as already demonstrated by studies such as Kuschyk et al. [11]. Secondly, the ability to non-invasively monitor these changes using NICaS^®^ presents a significant advancement in patient care, enabling clinicians to make informed decisions without the risks of invasive techniques. This is especially beneficial in high-risk populations, such as elderly patients or those with multiple comorbidities, where invasive procedures pose additional complications. Nevertheless, this study may serve as a basis for generating hypotheses, with its findings warranting confirmation in future randomized clinical trials. Moreover, the small sample size (eight patients) limits the generalizability of the findings. Larger, randomized controlled trials with extended follow-up periods are needed to confirm these results. In conclusion, we demonstrated that CCM significantly improves SV and TPRI in heart failure patients, with measurable hemodynamic changes occurring as early as one week post-implantation. The use of the NICaS^®^ system for non-invasive monitoring offers a reliable and safe method for tracking these changes in real time. Future research should focus on larger populations and longer-term follow-up. 

## Figures and Tables

**Table 1:** Patients’ Characteristics.

LVEF	Sex	Cardiomyopathy	Comorbidities	Therapy
28	Male	Dilatative	Chronic kidney disease Pulmonary nodule under follow-up	Omeprazole Aspirin (Low dose) Carvedilol Furosemide
45	Male	Dilatative	Permanent Atrial Fibrillation Type 2 Diabetes Mellitus Obesity Obstructive Sleep Apnea syndrome (OSAS)	Pantoprazole Rivaroxaban Sacubitril-Valsartan Bisoprolol Furosemide Spironolactone Empagliflozin
43	Male	Dilatative	Type 2 Diabetes Mellitus	Sacubitril-Valsartan Bisoprolol Furosemide Spironolactone Empagljflozin
33	Female	Dilatative	Atrial Fibrillation Hyperhomocysteinemia Chronic Obstructive Pulmonary Disease (COPD) Anxiety Depressive Disorder Bronchopneumonia focus	Pantoprazole Sacubitril-Valsartan Apixaban Clopidogrel Metoprolol Ivabradine Furosemide Spironolactone Rosuvastatin Ezetimibe Folic Acid Fluticasone/Vilanterol Empaglifozin
40	Female	Dilatative	Diabetes Obesity Hyperuricemia	Pantoprazole Sacubitril-Valsartan Apixaban Clopidogrel Metoprolol Ivabradine Furosemide Spironolactone Empaglifozin
31	Male	Ischemic	Arterial Hypertension Dyslipidemia Diabetes Mellitus Chronic Kidney Disease	Pantoprazole Spironolactone Bisoprolol Clopidogrel Allopurilnol Empaglifozin Ivabradine Ezetimibe/Atorvastatin Furosemide Sacubitril-Valsartan Linagliptin Dapaglifozin Ibandonatre (Neodidro)
36	Male	Ischemic	Normochromic-normocytic Anemia Diabetes Mellitus Hyperuricemia Previous Transient Ischemic Attack Carotid Atheromatosis	Pantoprazole Clopidogrel/Aspirin Furosemide Spironolactone Sacutril/Valsartan Carvedilol Ranolazine Allopurinol Ezetimibe/Atorvastatin Empaglifozin
33	Male	Ischemic	Chronic Kidney Disease Aneurysm treated with Endovascular Aneurysm Repair Arterial Hypertension Dyslipidemia	Omeoprazol Metoprolol Amiodarone Furosemide Clopidogrel Aspirin Febuxostat Valsartan Atorvastatin Empaglifozin

**Table 2:** Results of the Comparison Between Distributions at Different Time Points Using the Mann–Whitney Test and the Kolmogorov–Smirnov Test.

Comparison	Mann–Whitney		Kolmogorov–Smirnov	
	U	*p*-Value	D	*p*-Value
One Week–Baseline	1377	0.002	0.2969	0.0071
One Month–Baseline	1014	<0.001	0.5	<0.0001
Three Months–Baseline	752	<0.001	0.5625	<0.0001
One Month–One Week	1384	0.0014	0.3750	0.02
Three Months–One Week	999	0.0014	0.4844	<0.0001
Three Months–One Month	1377	0.0013	0.3281	0.002

## Data Availability

The original contributions presented in this study are included in the article. Further inquiries can be directed to the corresponding author.

## Abbreviations

The following abbreviations are used in this manuscript:

HF	Heart Failure
NYHA	New York Heart Association
CRT	Cardiac Resynchronization Therapy
LV	Left Ventricular
ICD	Implantable Cardioverter Defibrillator
ESC	European Society of Cardiology
CCM	Cardiac Contractility Modulation
SV	Stroke Volume
TPRI	Total Peripheral Resistance Index
CO	Cardiac Output
SVR	Systemic Vascular Resistance
BNP	B-type natriuretic peptide
LVEF	Left Ventricular Ejection Fraction
OMT	Optical Medical Therapy
